# Œdopsophia, or Noisy Expulsion of Gas from the Vagina

**Published:** 1844-01

**Authors:** 


*Œdopsophia*, or *Noisy Expulsion of Gas from the Vagina.* This affection occurs in women (especially lame women) whose inferior vulvar aperture is narrow. Oftentimes no symptom precedes it, and the patient is not aware of the existence of the complaint until the noisy expulsion occurs. The distended state of the parts may be felt by examination by the rectum, and the gas in part expelled by pressing on the hypogastrium. When the affection arises from a foreign body in a state of putrefaction, it will cease on the removal of this; but in instances where none such existed, Lisfranc has frequently rendered the patient's condition much more supportable, by instructing her, prior to going into society, or, under any circumstance where the unexpected expulsion would prove annoying, to secure herself from this, by introducing a finger into the inferior aperture of the vulva, and thus give vent to it. In examining females for other complaints, he has frequently detected this affection, although concealed by the patients, and has always succeeded in affording great comfort by the above suggestion. If the vagina is capable of secreting this gaseous substance, it is from its being in a condition of inflammation or irritation, and therefore means of a mild antiphlogistic character, modified by circumstances, must be had recoursə to, *e. g.*, venesection, warm baths, lavements, &c.—*Med. Chir. Rev.*, *Oct.*, 1843, from *Lisfranc's Clinique Chirurgicale de l'Hôpital de la Pitie.*

